# Surgical Management of Valvular Heart Disease in Mucopolysaccharidoses: A Review of Literature

**DOI:** 10.3390/biomedicines10020375

**Published:** 2022-02-04

**Authors:** Barbara A. Rosser, Calvin Chan, Andreas Hoschtitzky

**Affiliations:** 1 Department of Congenital Heart Surgery, Royal Brompton Hospital, London SW3 6NP, UK; calvin.chan4@nhs.net (C.C.); a.hoschtitzky@rbht.nhs.uk (A.H.); 2 Department of Surgery and Cancer, Imperial College London, St. Mary’s Hospital, London W2 1NY, UK

**Keywords:** mucopolysaccharidosis, structural heart disease, aortic valve, mitral valve, glycosaminoglycans, valve replacement

## Abstract

Mucopolysaccharidoses are extremely rare diseases that are frequently presenting with structural heart problems of the aortic and mitral valve in combination with myocardial dysfunction. In a substantial proportion, this leads to heart failure and is a leading cause of death in these patients. As this glycosaminoglycan degradation defect is associated with other conditions strongly influencing the perioperative risk and choice of surgical technique, multidisciplinary planning is crucial to improve short- and long-term outcomes. The extensive variance in clinical presentation between different impaired enzymes, and further within subgroups, calls for personalised treatment plans. Enzyme replacement therapies and bone marrow transplantation carry great potential as they may significantly abrogate the progress of the disease and as such reduce the clinical burden and improve life expectancy. Nevertheless, structural heart interventions may be required. We reviewed the existing literature of the less than 50 published cases regarding surgical management, technique, and choice of prostheses. Although improvement in therapy has shown promising results in protecting valvar tissue when initiated in infancy, concerns regarding stability of this effect and durability of biological prostheses remain.

## 1. Introduction

Mucopolysaccharidoses (MPS) describe a family of rare inherited lysosomal storage diseases characterised by specific deficiencies in lysosomal enzymes necessary for the degradation of glycosaminoglycans (GAGs). Despite cardiac involvement being common in MPS, leading to structural heart disease in 49–90% of cases [1], current evidence for surgical management in this cohort is limited to approximately three-dozen case reports. This review discusses the existing literature, focussed on surgical considerations, therapy options including choice of prostheses, timepoint of intervention, and results in patients with this rare disease.

## 2. Incidence

MPS subtypes are differentiated biochemically by associated lysosomal enzyme deficiency and resulting GAG accumulation. Currently, seven distinct types of MPS have been identified (Table 1); MPS III and IV are comprised of four and two more subtypes. Additionally, MPS I is classified into three subtypes, representing the spectrum of severity of the clinical manifestation (Hurler syndrome [most severe], Hurler-Scheie syndrome, and Scheie syndrome [least severe]) [2]. With the exception of MPS II (X-linked recessive), all MPS follow an autosomal recessive inheritance pattern [1].

The cumulative global incidence of MPS is estimated to be 1.04–16.9 cases per 100,000 live births [3]. The incidence of MPS types differs geographically, suggesting regional or ethnic variations [2]. In Europe, the cumulative birth incidence is estimated to be 3.14/100,000 [3]. As the clinical manifestation including age at symptom onset vary greatly between individuals and types of MPS, diagnosis can be challenging [4]. Additionally, dedicated MPS new-born screening programmes are not yet broadly available; evidence from small-scale screening programmes suggests that the incidence of MPS is likely to be much higher than currently reported, for example MPS I screening in a cohort in Taiwan was found to indicate an incidence of 5.66/100,000 [5,6].

A broad variation of clinical presentation exists even within MPS types and subtypes [7]. The course of MPS can be severe, leading to death in childhood or adolescence. Conversely, patients with attenuated forms of MPS may be able to achieve near normal life expectancy, especially since the introduction of enzyme replacement therapies and bone marrow transplantation. A case of two female patients with MPS IV-A, aged 74 and 70, has recently been reported [8]. It can be expected that ERT will influence the occurrence of structural heart disease in the cohort towards later presentation, conversely may lead to an increase in absolute numbers as life expectancy increases.

**Table 1:** Overview of types of mucopolysaccharidoses. Incidence figures compiled from Khan 2017 [3].

MPS Type	Name	Deficient Enzyme	Glycosaminoglycan	Incidence per 100,000	Typical Age of Diagnosis (Years)	Typical Pre-treatment Life Expectancy (Years)	Structural Heart Disease (%)
I-H	Hurler syndrome	α-L-iduronidase	Heparan sulfate Dermatan sulfate	0.11–3.62	1 [9]	8 [10]	49 [9]
I-HS	Hurler-Scheie syndrome				4 [9]	21.6 [10]	59 [9]
I-S	Scheie syndrome				9.4 [9]	Normal [11]	68 [9]
II	Hunter syndrome	Iduronate sulfatase	Heparan sulfate Dermatan sulfate	0.1–2.16	4.2 ± 4.2 [12]	13.4 [13]	57 [12]
III-A	Sanfilippo syndrome A	Heparan sulfamidase	Heparan sulfate	0.26–1.89	4.9 ± 4.4 [14]	15.2 ± 4.2 [15]	50 [16]
III-B	Sanfilippo syndrome B	N-acetylglucosaminidase			4.9 ± 4.5 [14]	18.9 ± 7.3 [15]	
III-C	Sanfilippo syndrome C	Heparan-α-glucosaminide N-acetyltransferase			12.0 ± 6.5 [14]	23.4 ± 9.5 [15]	
III-D	Sanfilippo syndrome D	N-acetylglucosamine 6-sulfatase			8.2 ± 5.2 [14]	Unknown	
IV-A	Morquio syndrome A	Galactose-6-sulfate sulfatase	Keratan sulfate Chondroitin sulfate	0.09–3.62	4.7 [17]	25.0 ± 17.4 [18]	50 [19]
IV-B	Morquio syndrome B	β-galactosidase	Keratan sulfate		Unknown	Unknown	Unknown
VI	Maroteaux–Lamy syndrome	N-acetylgalactosamine-4-sulfatase	Dermatan sulfate	0.0132–7.85	7 ± 7.8 (range 0–55) [20]	Rapid: 20–30 Slow: 40–50 [21]	90 [20]
VII	Sly syndrome	β-glucuronidase	Heparan sulfate Dermatan sulfate Chondroitin sulfate	0.038–0.29	0.9 [22]	Infancy–50 [22]	50 [23]
IX	Natowicz syndrome	Hyaluronidase	Hyaluronic acid	Sporadic	Unknown	Unknown	Unknown

Data displayed as mean ± standard deviation or median.

## 3. Pathophysiology

The deficiency or malfunction of enzymes present in MPS are insufficient for a correct degradation of glycosaminoglycans, long linear polysaccharides with highly hydrophilic characteristics produced in the Golgi apparatus or by integral membrane synthases. This disturbance in the catabolism leads to intra-lysosomal, thus intracellular accumulation of GAG in the cardiac tissues [11,24]. Dermatan sulphate is regularly found in heart valves, tendons, blood vessels, pulmonary tissue and skin, and a disbalance of this type of GAG is especially associated with structural heart disease, which is reflected by higher occurrence of structural heart disease in MPS types I, II, VI, more than types III and IV [25].

### 3.1. Valve Disease

Progressive cardiac valvular disease is a prominent cardiac feature of MPS and was first described in 1960 [26]. Although all cardiac valves may be affected, left-sided valve issues are the most common (Table 2) with mitral valve and aortic valve being the predominantly affected structures [11]. Further, these abnormalities can present as combined, complex structural disease and may additionally be associated with left ventricular hypertrophy [27].

In valvular interstitial cells, which normally are responsible for valve growth and repair, MPS leads to activation and development of large, GAG-laden “Hurler” cells [28]. Additionally, an inflammatory and macrophage response is induced, causing further tissue dysfunction [29]. These GAG-triggered processes lead to valve thickening, diminished leaflet mobility, and coaptation deficiency. Valve leaflet thickening has been reported to affect 60–90% of MPS patients [27]. Severe cases can also present with shortened chordae tendineae [30]. The combination of these processes can lead to mitral regurgitation, mitral stenosis, aortic regurgitation and aortic stenosis and combinations. Increased dermatan sulphate accumulation further is associated with myxomatous mitral valve degeneration [31].

**Table 2:** Cardiac abnormalities in children and adults with MPS from reported studies.

MPS Type	Study	No. Patients	Age Range	MV Abnormalities			AV Abnormalities			LVH (%)
				MR (%)	MS (%)	MVT (%)	AR (%)	AS (%)	AVT (%)	
I-H	Mohan 2002 [32]	29	1–24	38	10	24	7	3	14	31
I-S	Thomas 2010 [33]	50	1.8–62.9	76	32	ns	56	36	ns	Ns
II	Schwartz 2007 [34]	38	0.2–53	63	3	37	36	5	29	11
III	Wipperman 1995 [27]	30	1.3–16.4	47	0	83	30	3	40	Ns
IV-A	Harmatz 2013 [35]	325	1.1–65.6	25	16	ns	19	5	ns	1
VI	Kampmann 2014 [36]	37	0–42	M: 20 F: 22	M: 27 F: 15	ns	M: 36 F: 39	M: 7 F: 5	ns	M: 53 F: 27

Abbreviations: AR = aortic regurgitation; AS = aortic stenosis; AV = aortic valve; AVT = aortic valve thickening; LVH = left ventricular hypertrophy; MPS = mucopolysaccharidoses; MR = mitral regurgitation; MS = mitral stenosis; MV = mitral valve; MVT = mitral valve thickening; ns = not stated.

### 3.2. Myocardium and Large Vessels

The intracellular accumulation of GAGs leads to formation of large Hurler cells containing clear vacuole of mucopolysaccharides which also can be found in the myocardium. The triggered inflammatory response leads to increased collagen reaction of these cells with corresponding tissue fibrosis, thus, leading to a restrictive impaired ventricular function, predominantly in the left ventricle. The resulting echocardiographic findings besides diastolic dysfunction are left ventricular hypertrophy and left ventricular remodelling [36]. The structural changes in the myocardium further can lead to alteration in the conduction system clinically presenting with progressive AV block.

The same Hurler cells can be found in large vessels. Frequent concomitant appearances are enlarged aorto-ventricular junction, valsalva and sino-tubular junction, which is found in 30–41% of patients with MPS [37].

### 3.3. Coronary Artery Disease

In addition to valvular and myocardial infiltration, deposition of GAGs can also occur in the vascular smooth muscle cells, leading to diffuse, concentric narrowing and consecutive stenosis of the coronary arteries, and additional intima hypertrophy has been described [38]. Consequently, an accelerated course of coronary artery stenosis possibly leading to ischaemic heart failure has been reported to occur in MPS patients. Clinical symptoms of this process may remain absent until a later stage. Contradictory reports on association with different types of MPS have been published. While historically coronary artery disease is known to be associated with MPS I, one report found histopathological alterations predominantly in non-Hurler MPS [39]. Even more, exclusion of coronary artery disease is recommended prior to surgery [40].

## 4. Systemic MPS Treatment and Cardiac Disease

### 4.1. Haematopoietic Stem Cell Transplantation

First performed in patients with MPS I-H in 1981, haematopoietic stem cell transplantation (HSCT) is considered standard of care for these patients [41]. HSCT has been shown to improve symptoms in MPS I-HS, II, IV-A, VI, and VII, though there are high associated risks, particularly from graft-versus-host disease [42]. Therefore, HSCT is generally reserved for the more severe forms of MPS.

Despite clear improvements in life expectancy and neurosensory outcomes post-HSCT, long-term follow-up has revealed that approximately one-third of MPS I-H patients still showed progressive mitral and aortic insufficiency despite HSCT [43]. Thus, valve disease remains a significant disease burden despite treatment.

### 4.2. Enzyme Replacement Therapy

Enzyme replacement therapy (ERT) involves delivery of an exogenous analogue of the deficient enzyme through regular lifelong intravenous infusions. Currently approved ERTs include: laronidase (MPS I), idursulfase (MPS II), elosulfase alfa (MPS IV-A), galsufase (MPS VI), and vestronidase alfa (MPS VII) [44]. ERT significantly reduces urinary GAGs, a marker of MPS disease activity, and improves life expectancy and physical functioning [45,46,47]. Cardiac benefits of ERT, including improvements in ventricular hypertrophy and ejection fraction, have been noted [44,48,49].

However, the efficacy of ERT in stabilising valve disease in the MPS types is not as apparent. It is postulated that the avascularity of the cardiac valves diminishes the efficacy of ERT, making it ineffectual in halting the progression of valve disease [48]. Additionally, an antibody response to ERT has been shown to occur in some patients, reaching levels that could potentially affect the efficacy of ERT [50].

Ten-year follow-up of MPS I patients on ERT revealed that although two-thirds of patients achieved stabilisation of valve disease, approximately one-third of patients still had valvular deterioration [45]. Progression of mitral valve disease has also been noted in 10-year follow-up of MPS II patients on ERT [51]. Three-to-six-year follow-up in MPS IV-A patients suggested that ERT has little effect on valve function despite improvements in ventricular hypertrophy [19]. Finally, a two-year follow up of MPS VI patients showed significant progression in AR and no improvement in valve stenosis despite ERT treatment in the subset of patients who began ERT after childhood [48]. Current evidence suggests that ERT begun in infancy has a protective effect on valvar tissue [52,53,54] compared to treatment begun later in childhood [55,56,57]. However, the durability of these results has yet to be confirmed. As valvular dysfunction remains present and, in some cases, progressive despite ERT in patients across all MPS types, structural heart surgery eventually has to be considered.

### 4.3. Gene Editing Therapy

A variety of gene transfer and gene therapy approaches are currently in preclinical and clinical phase with 17 registered clinical trials, of which some have completed treatment with successful first safety endpoints and now progressed to a long-term follow-up phase. Further investigations on their clinical efficiency are to be expected.

## 5. Perioperative Management

### 5.1. Pre-Operative Considerations

As a rare, complex multisystemic disease, surgery should be performed in specialist centres experienced with MPS patients. A well-planned multidisciplinary approach is necessary with experienced anaesthetic input and a joint cardiac disease multidisciplinary team meeting is recommended. The potential risks of the procedure and anaesthesia should be weighed against the benefits and extensively discussed with the patient and relatives [58].

Other considerations include pre-operative ENT examination with naso-endoscopy or MRI assessment of the spine, brain, and airways. Additionally, coronary angiography should be performed prior to any cardiac procedure due to the increased prevalence of diffuse coronary disease in MPS patients [40].

### 5.2. Respiratory Tract

Respiratory symptoms are apparent across all MPS types and are important drivers of mortality [13]. Besides upper and lower airway obstruction, predisposing patients to frequent respiratory tract infections and obstructive symptoms [14], restrictive lung disease is also common, resulting from MPS manifestations such as a small thoracic cage, kyphoscoliosis, pectus carinatum, and displacement of the diaphragm superiorly due to hepatosplenomegaly [15]. As this adds to the perioperative risk, a preoperative lung function study is essential prior to cardiac surgery.

### 5.3. Cardiac Diagnostics and Symptom Assessment

It is important to understand that a large proportion of patients with MPS are limited in their exercise capacity due to musculoskeletal or spinal issues with a substantial number being wheelchair-bound. Therefore, it is often so that they only become symptomatic with advanced valve, myocardial or coronary artery disease. It is, therefore, important to closely monitor these patients in the cardiology clinic for subclinical advanced heart disease. MPS not only affects the heart valves, it also can lead to ventricular dysfunction with fibrosis, coronary artery disease, and electrophysiological symptoms. These three aspects of cardiac affection in MPS patients are important when planning a structural procedure, as concomitant severe coronary artery stenosis or restrictive left ventricular function will strongly influence the perioperative risk. Aortic abnormalities can also occur with MPS with a risk of dissection. Routine investigations with echocardiography and ECG should be standard of care. In situations where it is felt that the heart disease has advanced significantly so, detailed investigations are indicated. These include transthoracic echocardiography, coronary CT and CT angiography, ECG, and, if possible, cardiac magnetic resonance scanning. Coronary artery disease in these patients does not usually mimic atherosclerotic patterns, and, therefore, it is more useful to perform functional studies such as stress-echocardiography or myocardial perfusion scanning. Multidisciplinary discussions regarding consideration of intervention or surgery should always take place.

### 5.4. Anaesthetic Considerations

Abnormal anatomical features and tissue morphology due to GAG accumulation can pose challenges regarding airway control, intubation, intravenous access, and cervical spine stability. Although surgery is frequent in MPS (MPS I 81% at least one procedure) [59], patients are at increased risk when undergoing general anaesthetics procedures. Difficult and failed intubations are common [60] and should be carried out by experiences anaesthetists familiar with MPS patients with adequate intensive care and ENT backup.

Intubation and airway difficulties are due to narrowing of the upper airways with thickening of laryngopharyngeal structures, including macroglossia, tonsillar enlargement, and narrowed nasal passages [61]. Lower airway stenosis can involve malformed tracheal cartilage, abnormal vocal cords and airway oedema from recurrent respiratory infections [62]. Additionally, the short neck, raised maxilla, and small mouth opening due to stiff temporomandibular joints may make laryngoscopy difficult [61].

Additionally, cervical spine stability should be considered. Particularly in MPS IV, patients can present with odontoid hypoplasia, which is a considerable risk factor for atlantoaxial instability. If not properly managed, this can result in spinal cord compression, eventually leading to paralysis or death [63]. Therefore, minimising head and neck movement by manual stabilisation is advised during induction and intubation [58]. The head should maintain a neutral position; this can make direct laryngoscopy difficult. Thus, fibreoptic intubation, or an angulated video laryngoscope may be used [64].

### 5.5. Post-Operative Considerations

Careful post-operative planning is crucial; there is an increased rate of post-operative mortality with MPS patients [59]. Intensive care unit admission is necessary; extracorporeal membrane oxygenation or intra-aortic balloon pump therapy may be required for post-operative ventricular dysfunction [65]. Extubation should be performed only when the patient is fully conscious and coughing [58]. Post-extubation laryngeal oedema can occur, and steroid administration before planned extubation has been suggested to reduce this complication [66,67]. Additionally, steroid cream can be applied to reduce oral mucosal and tongue swelling [58]. In case of serious airway concerns, post-operative elective tracheostomy may has to be considered; this should be discussed with the patient pre-operatively [68]. Furthermore, precautionary equipment for emergency tracheostomy and fibreoptic reintubation should be available at extubation should complications arise [66].

## 6. Procedure Considerations

### 6.1. Valve Replacement

#### 6.1.1. Timing of Surgery

The best time point for heart valve replacement can be a challenging decision in MPS patient. Complicating factors, cardiac and non-cardiac MPS symptoms must be considered, best to be assessed in an interdisciplinary conversation considering all available medical history as well as recent cardiac and respiratory examinations. Clinical manifestation and progression show strong variation between different types of MPS, additionally individual differences between patients of the same type of the disease can be observed. Therefore, a personalised treatment plan is required for these patients.

#### 6.1.2. Prosthesis Type

Most reported valve replacements involved mechanical rather than bioprosthetic implants. This seems an obvious choice as mechanical valves are preferable in the relatively young MPS patient population; bioprosthetic prostheses have a limited durability due to leaflet deterioration, particularly true for younger patients [69]. Additionally, it is possible, given the pathophysiology of MPS, that bioprosthetic degeneration could be affected by GAG deposits leading to even quicker degeneration of the bioprosthetic leaflets. On the other hand, mechanical valve replacement requires patients to be on full anticoagulation with associated annual significant complication incidence of 1–2% in a non-MPS population, which conceivably could be higher in the MPS population.

Although current literature suggests mechanical valves as implants, under certain circumstances biological prostheses might be considered. Increasing experience in new therapy options such as ERT and optimised treatment protocols have the potential to enable prosthesis degeneration similar to that of non-MPS patients. This is particularly true for patients presenting at later stages in life, thus milder forms of MPS. For patients with neurological impairment due to MPS, or other reasons of impaired suitability for oral anticoagulation, biological implants might be considered. Furthermore, the possibility of an interventional valve-in-valve implantation has the potential to prolong the interval to reoperation in cases where a sufficiently sized valve can be used, as also mentioned by Dostalova et al. [70,71]. Most centres treating these complex patients would still shy away from using non-mechanical valve substitutes at present, due to a lack of supportive data for this strategy.

Regarding the effect of ERT on progress of natural valve changes, controversial experiences and considerations have been reported. During an elective aortic valve replacement, despite significant MS on echocardiography, Torre et al. elected not to perform double valve replacement on a 40-year-old MPS VI patient. The authors reasoned that, since the patient was on ERT for three years, the MS would not progress further [57]. Additionally, the patient presented with a small mitral annulus, which would warrant either an annular enlargement or a small prosthetic valve. The former is complicating the procedure significantly with corresponding effect on risk and morbidity, whereas the latter incurs risk of patient-prosthesis mismatch, full anticoagulation, and residual gradient. On the other hand, the patient was at risk of having to undergo a further valve replacement procedure, should the mitral valve disease progress. This scenario has been highlighted in several reports where progressive valve disease has led to a second valve replacement operation despite ERT [72,73]. Given the variable rate of disease progression and response to ERT between patients, deciding between conservative or aggressive valve replacement can be difficult. Risks and benefits of both courses of treatment should be discussed in a multidisciplinary setting and with patients. Further long-term analysis of valve disease progression under ERT is expected to provide further evidence. An overview on double valve replacement reports can be found in Appendix A Table A2.

The systemic nature of MPS can often cause multi-valvular incompetence. Poor annular tissue quality, often described as “friable”, “hard”, and “not pliable”, has been reported to complicate valve implantation in MPS patients [56,65,74,75]. Kitabayashi et al. suggested the use of an equine pericardial patch between the atrial wall and the prosthetic ring to reinforce the suture line [65]. Similarly, Bell et al. found anchoring of the prosthesis on the annular tissue challenging and suggested the use of felt pledgets to reinforce the prosthetic sutures [56]. These adjuncts may aid in preventing valve dehiscence and paravalvular leakage, thereby reducing the risk of valve failure and redo surgery.

### 6.2. Aortic Valve

Regarding aortic valve replacement, one of the first MPS patients to undergo a mechanical valve replacement received a Braunwald–Cutter prosthesis and was reported to be well 19 years later, having undergone further elective aortic and mitral valve replacements [76,77]. Similar to the mitral valve, a majority of cases reported difficulties with small aortic root and annuli (Appendix A Table A1).

Nick’s technique for root enlargement has been used successfully in several MPS patients [76], this is done by extending the inferior aspect of the aortotomy incision through the aortic annulus into the base of the anterior mitral leaflet. A teardrop shaped Dacron or bovine pericardial patch is used to enlarge the incision. The prosthetic aortic valve is then sutured into the enlarged annulus, with a segment attached to the patch.

Konno aorto-ventriculoplasty with root enlargement has also been reported in at least one case [72]. While this procedure offers the possibility of an extended root enlargement with an annular increase of 3–4 mm, it is a complex procedure involving creation of a ventricular septal defect and a double-patch closure [78]. The choice of method for root enlargement should be dictated by surgical experience and overall patient risk [79].

#### 6.2.1. Ross Procedure

The Ross procedure can bring the advantage of not requiring anticoagulation and expected good long-term freedom from reintervention in non-MPS patients (81% at 15 years) [80]. Two documented Ross procedures have been performed on MPS patients, both of which involved post-operative diagnoses of MPS (Table 3). One case reported visual loss due to optic nerve ischemia occurring three days post-op in a 15 year-old MPS II patient [81]. Stable cardiac function was reported at two-year follow-up despite refusal of ERT. In the second case, a MPS IV-B patient re-presented with severe autograft regurgitation and severe pulmonary homograft calcific stenosis twelve years after the procedure [82]; the patient died several days later. It is thought that GAG deposition in both the native autograft and already calcification-prone homograft precipitated the accelerated course of valve failure. Thus, due to risk of rapid valve degeneration, the Ross procedure should not be considered for MPS patients.

#### 6.2.2. Aortic Valve Repair/Ozaki Procedure

When considering the disappointing results with the Ross procedure in these patients, as well as progression of valve disease under ERT, there is scanty support for aortic valve repair, and none for complex reconstruction such as the Ozaki procedure in MPS patients.

#### 6.2.3. Transcatheter Aortic Valve Implantation (TAVI)

In the wider patient population, similarity in mid-term outcomes between TAVI and surgical aortic valve replacement has previously been suggested [83,84]. A successful TAVI (Edwards-Sapien 26 mm) was performed on a MPS I-S patient in 2014 with reported rapid recovery and resolution of symptoms (Table 4) [85]. However, given that TAVI valves are bioprosthetic and constructed with bovine or porcine material, the durability of these valves in MPS patients is currently unknown. There is one case report describing redo surgical aortic valve replacement in a 54 year old female patient with MPS of undescribed type two-and-a-half years after TAVI [86]. This was due to severe AS resulting from structural valve deterioration thought to be associated to GAG deposition. The patient unfortunately died of a “stuck mitral valve” nine days post-redo surgery. However, in patients with very high surgical risk due to advanced MPS or other reasons, TAVI can represent a treatment option in case a surgical replacement is not considered feasible. There would still be significant concerns regarding vascular access routes for these patients if so contemplated.

### 6.3. Mitral Valve

Regarding mitral valve replacement, the majority of cases in literature have reported challenges pertaining to small mitral annuli. Implantation of an appropriate valve size for body size may be difficult and, thus, prosthesis–patient mismatch may occur. As we know, this is associated with poor haemodynamic function, higher risk of cardiac events and increased mortality [87]. Therefore, with a small diameter annulus, it is especially important to select a prosthesis that provides the largest possible effective orifice area to minimise the postoperative pressure gradient across the valve [88]. Should the mitral annulus be too small to fit a mitral prosthesis, an reversely implanted aortic valve prosthesis needs to be considered [76]. However, an enlargement of the mitral annulus might be a superior technique for these cases as to minimise transvalvular gradient as well as improved valve durability in biological prosthesis of larger sizes [56,89]. It needs to be taken into context though of the small body size area and reduced activity levels that many of these patients can achieve though. A summary of published mitral valve replacements is given in Appendix A Table A3.

#### Mitral Valve Repair

Successful repair of the mitral valve in MPS patients has only been reported twice. Once in a 6-year-old MPS III patient and with very limited follow-up of one year. The repair comprised chordal shortening and a limited annuloplasty with pledget supported mattress sutures in both commissures [90]. Albeit a progression of the local disease must be expected as the clinical presentation already affected the valve tissue, it might be considered as bridging therapy to allow somatic growth facilitating a larger prosthesis at a later timepoint. It should remain a niche indication reserved for situations where no prosthetic valve is anatomically fitting. However, in case ERT, bone marrow transplantation or other treatments in the future are found to significantly slow down disease progression in milder forms of MPS, a mitral valve repair in patients presenting in adulthood might become more commonly considered. Further long-term results of ERT involving histopathological assessment would be required for this.

## 7. Conclusions

Surgical valve replacement is often required for the progressive, severe valvular disease that can occur in MPS patients despite advances in systemic treatment. Challenges associated with valve implantation include poor tissue quality and small valve annuli, which may necessitate annular enlargement or a small-diameter mechanical valve both for aortic and mitral position. Additionally, perioperative risks are increased in this cohort of patients, in particular due to difficulties with airway control and ventricular function. Careful multidisciplinary pre-operative planning is essential, personalised solutions for this broad spectrum of clinical presentation are necessary and procedures should be carried out by a clinical team experienced in managing MPS patients. The effect of ERT both on native valve tissue as well as bioprosthetic tissue is yet to be further investigated.

## Figures and Tables

**Table 3:** Description of case reports of Ross procedures in patients with MPS.

Case	Type	Age/Sex	Presentation	Pre-op Findings	Procedure	Surgical Notes	Outcome	Author Comments
Curran (2019) [81]	II	15 M	Elective procedure; Asymptomatic on ACEi	Mild AS, severe AR; Dilated LV; Moderate systolic dysfunction	Ross procedure: 24 mm pulmonary homograph	Uneventful procedure	Visual loss 3d PO: NAION; PO diagnosis of MPS II; Stable cardiac status, vision improvement at 2 yr F/U	- NAION encountered post-cardiac surgery. - 20–40% show long-term improvement. - It is unknown if MPS contributes to NAION.
Barry (2006) [82]	IV-B	32 F	Dysponea on exertion	Bicuspid AV, severe AR; LV enlargement	Ross procedure: 29 mm pulmonary homograph	AV thickened with retraction of leaflets	PO sternal wound infection; Severe autograft regurgitation at 12 yr F/U; Severe pulmonary homograft stenosis; Died aged 44	- Autograft failure due to MPS IV valvular degeneration. - Collagen abnormalities and KS deposition affected the autograft. - KS deposition in calcific prone pulmonary homograft may accelerate stenotic process. - MPS is contraindication to Ross procedure.

Abbreviations: ΔP = mean transvalvular pressure gradient; ACEi = Angiotensin-converting-enzyme inhibitor; AR = aortic regurgitation; AS = aortic stenosis; AV = aortic valve; F/U = follow-up; KS = keratan sulfate; LV = left ventricle; MPS = mucopolysaccharidoses; NAION: non-arteritic anterior ischemic optic neuropathy; PO = post-operative.

**Table 4:** Description of the case report of transcatheter aortic valve implantation in a patient with MPS.

Case	Type	Age/Sex	Presentation	Pre-op Findings	Procedure	Surgical Notes	Outcome	Author Comments
Felice (2014) [85]	I-S	30 M	Dyspnoea and exertional syncope	Thickened AV leaflets. AS: 0.8 cm^2^, ΔP 36 mmHg; LVH: 13 mm	TAVI: Edwards Sapien XT 26 mm	Elective tracheostomy; No surgical complications	Rapid recovery and resolution of symptoms	- TAVI chosen due to patient concerns about perioperative risks and need for anticoagulation. - Durability of biological and TAVI valves are unknown.

Abbreviations: ΔP = mean transvalvular pressure gradient; AS = aortic stenosis; AV = aortic valve; LVH = left ventricular hypertrophy; TAVI: transcatheter aortic valve implantation.

## Data Availability

Not applicable.

## Appendix A

**Table A1:** Description of case reports of aortic valve replacement in MPS patients.

Case	Type	Age/Sex	Presentation	Pre-op Findings	Device/other Procedures	Surgical Notes	Outcome	Author Comments
Masuda (1993) [91]	I-S	62 M	General fatigue	Severe AS, mild AR; Slight MV thickening	Bjork–Shiley	Diffuse AV calcification and thickening; Small annulus	Uneventful F/U	- Calcified aortic annulus made resection difficult. - No issues with valve insertion.
Pagel (2009) [63]	IV-A	31 F	Progressive exertional dyspnoea, fatigue	AV thickening and sclerosis, severe AR; MV thickening, mild MR; EF 45%	27 mm SJM	Awake oral fibreoptic intubation; Thickened aortic valve	D/C 5 d PO	- Odontoid hypoplasia can cause cervical-occipital dislocation or cord compression during direct laryngoscopy. - Patient’s brother had AVR and sustained atlantoaxial-occipital subluxation and quadriparesis.
Dostalova (2018) [70]	IV-B	60 F	Dyspnoea NYHA II-III	AS: 0.45 cm^2^; LVH; Mild LVOTO	No. 19 Carpentier–Edwards bioprosthesis; Septal myectomy	Uneventful operation	No dyspnoea, mild MR and TR on 3 yr F/U	- Septal myectomy permits TAVI in the future if bioprosthetic malfunctions.
Wilson (1980) [77]	VI	43 M	Angina pectoris	Severe AS with mild AR; MV thickening, mild MS	A-22 Braunwald–Cutter; MV commissurotomy	LVH; AV severely stenotic and moderately calcified; MV leaflet fusion, thickened chordae	Asymptomatic on 19 yr F/U	- No unusual technical difficulties encountered. - No periprosthetic leakage.
Torre (2016) [57]	VI	40 F	Dyspnoea; On ERT	Severe AS: 0.76 cm^2^, ΔP 76 mmHg, moderate AR; Severe MS: 1.5 cm^2^, ΔP 15 mmHg; Mild LVH; EF 65%	19 mm mechanical prosthesis	Severe AV degeneration and annular hypoplasia.	D/C 8 d PO; Asymptomatic NYHA I 2 yr later	- ERT may delay worsening valve disease so conservative treatment for MV.

Abbreviations: ΔP = mean transvalvular pressure gradient; AR = aortic regurgitation; AS = aortic stenosis; AV = aortic valve; AVT = aortic valve thickening; D/C = discharge; EF = ejection fraction; ERT = enzyme replacement therapy; F/U = follow-up; LVH = left ventricular hypertrophy; LVOTO = left ventricular outflow tract obstruction; MPS = mucopolysaccharidoses; MR = mitral regurgitation; MS = mitral stenosis; MV = mitral valve; MVT = mitral valve thickening; NYHA = New York Heart Association functional classification; PO = post-operative; SJM = St Jude Medical mechanical prosthesis; TAVI = transcatheter aortic valve implantation; TR = tricuspid regurgitation.

**Table A2:** Description of case reports of double valve replacement.

Study Type	Type	Age/Sex	Presentation	Pre-Op Findings	Devices/Other Procedures	Surgical Notes	Outcome	Author Comments
Goksel (2009) [92]	I	12 M	Symptoms of HF	AS: ΔP 50 mmHg; Severe MS: ΔP 14 mmHg	AV: 19 mm SJM MV: 25 mm SJM	Stenotic, thickened leaflets on both valves; Pledgetted 2-0 Ti-Cron everting sutures used; Mitral leaflets preserved.	D/C 7 d PO; NYHA I at 1 yr F/U; Normal valve function; No thromboembolic complications	- Use low-profile, bileaflet mechanical valves, as large as possible, to provide effective orifice area during growth. - Optimizing anticoagulation and monitoring is important.
Sato (2015) [55]	I-HS	56 F	AS and MS; NYHA III; On ERT	Severe AS and MS; LVH; PAP 43 mmHg	ns; CABG	Difficult intubation; Delayed extubation due to tracheal deformity.	Healthy since procedure	- Patient had degenerated valves despite ERT and lowered urinary GAG.
Robinson (2017) [93]	I-HS	39 F	Exertional dyspnoea	AS: 0.6 cm^2^, ΔP 70 mmHg; MS: 2.1 cm^2^, ΔP 6 mmHg	AV: 19 On-X MV: 25 On-X; Root enlargement with pericardial patch	ns	Died 17 d PO from haemorrhagic infarction; Ostium of RCA occluded by prosthesis cloth ring	- Calcified MV annulus makes valve implantation difficult. - Some patients require a root widening patch. - Difficult intubation which took 2.5 h to achieve.
Rocha (2012) [94]	I-HS	47 F	Exertional dyspnoea and palpitations	Thickened, calcified and restricted valves; AS: 0.8 cm^2^, ΔP 37 mmHg; MS: 1.6 cm^2^, ΔP 7 mmHg; EF 55%	AV: 19 mm SJM; MV: 25 mm SJM;	Uneventful.	D/C 6 d PO; Acquired Mediport line infection with 2 mm mass on MV; Redo double valve replacement and DeVega tricuspid annuloplasty for TR; Asymptomatic after 12mo	- Double-valve replacement reflects severe valve involvement of MPS I. - Presence of chronically implanted catheter may increase risk of infection.
Butman (1989) [95]	I-S	42 F	Chest pain and congestive HF admissions	Severe AS: 0.5 cm^2^, ΔP 100 mmHg; MS: 0.9 cm^2^, ΔP 16 mmHg	AV: 20 mm Medtronic Hall; MV: 25 mm SJM	Severe valve disease and calcification; Thickened chordae.	D/C 12 d PO	- Uneventful surgery. - Annuli were of sufficient integrity for good valve replacement.
Minakata (1998) [74]	I-S	52 M	Incidental LVH on ECG	AV moderately calcified and thickened; AS: 0.6 cm^2^, ΔP 46 mmHg; MV leaflets nodular, severely thickened; MS: 1.45 cm^2^, ΔP 7 mmHg; Severe LVH	AV: 19 mm SJM AHP; MV: 25 mm SJM	AV cusps calcified, thickened, hard and rolled free edge; MV leaflets scalloped, thickened; Fusion of commissures; Shortening of chordae.	IABP for low-output syndrome. No leak at 17 mo F/U	- Thickening of leaflets with restricted movement and hypertrophy typically observed in MPS. - Conscious intubation with aid of fibreoptic bronchoscope. - Difficult valve replacement due to small annulus. - Annulus enlargement may be required.
Murashita (2011) [66]	I-S	35 F	Severe AS and MS detected on F/U	AV annulus 16 mm; AS: 0.22 cm^2^; Severe MS: 0.81 cm^2^; subvalvular apparatus severely thickened; LVH; EF 50%; PAP 64 mmHg	AV: 16 mm ATS-AP; MV: 20 mm ATS-AP (inverted AV); Root enlargement with Hemashield Gold Dacron patch	Unsuccessful ET attempt; Fibreoptic intubation needed; Severely thickened leaflets, chordae and papillary muscle; MV annulus unclear; Hydrocortisone given for postextubational laryngeal oedema.	D/C 19 d PO. Good prosthetic and cardiac function	- Only small prosthetic valves can be implanted. - Aortic root enlargement may be required. - Nasotracheal intubation with fibreoptic intubation is a viable strategy. - Emergency tracheostomy should be at intubation and extubation. - Steroids before extubation could reduce laryngeal oedema.
Takahashi (2017) [96]	II	62 M	Exertional dyspnoea; On ERT	Thickened partially calcified MV; MS: 1.2 cm^2^, ΔP 8 mmHg; Moderate MR; Severely calcified AV; AS: 0.8 cm^2^, 45 mmHg; EF 60%; Severe TR; PAP 69 mmHg	AV: 20 mm ATS; MV: 27 mm SJM; TV annuloplasty with 28 mm Physio tricuspid ring	AV: severe calcification on cusps and annulus; MV: segments of posterior leaflet thickened and curled up.	D/C 15 d PO and doing well at 14mo F/U	- Degenerative valve changes more prominent in AV.
Demis (2021) [97]	VI	42 M	Symptomatic severe AS and moderate MR	Severe AS: 69 mmHg; Moderate MR: 0.37 cm^2^, regurgitant fraction 50%; LVH mass index 120 g/m^2^; EF 55%; PAP 28 mmHg	AV: Sorin Bicarbon Slimline 17; MV: Sorin Bicarbon Fitline 23; Modified Nick’s procedure with polyester vascular patch	ET intubation impossible even with video assistance; Intubation achieved with fibreoptic bronchoscope; Fibrotic and stenotic AV with small root and degeneration of MV leaflets.	D/C 8 PO; Asymptomatic and no valve leak at 18 mo F/U	- Dangerous anaesthetic induction. - Difficult sternal retraction due to skeletal abnormalities. - Small AV and MR annulus increases mismatch risk. - Anaesthetic and surgical preparation are mandatory.
Hachida (1996) [98]	VI	41 M	Progressive exertional dyspnoea; Severe peripheral oedema	AS: 0.34 cm^2^, ΔP 36.2 mmHg; AR Grade II; Thickened MV: 0.43 cm^2^; EF 49%.	AV: 19 mm SJM; MV: 25 mm SJM; TV: annuloplasty	Difficult intubation due to stiff joints and jaw; AV: degenerated and thickened cusps with commissural fusion; Small aortic annulus; MV thickened.	Extubated 4 d PO; EF 53%. D/C 45 d PO; Asymptomatic, ΔP 18 mmHg at 3 yr F/U	- Small, degenerative annulus could only fit a small valve prosthesis.
Tan (1992) [76]	VI	30 M	Exertional dyspnoea	AS with bicuspid AV: 0.43 cm^2^, ΔP 38 mmHg; MS with thick and stenotic leaflets: 0.74 cm^2^, ΔP 19 mmHg; EF 60%	AV: 19 mm SJM; MV: 21 mm SJM (inverted AV); Root enlargement with teardrop-shaped pericardial patch	Difficult intubation; Pt hypoxemic due to acute pulmonary oedema; Transnasal intubation with fibreoptic bronchoscopy; AV stenotic with three thickened cusps; MV thick leaflets, mild fusion.	Extubated 4 d PO; D/C 12 d PO; EF 72%. Tamponade at 5mo; Died of respiratory complications during knee arthroscopy procedure	- Difficulty inserting adult-sized prosthesis. - Nick’s technique used for root enlargement. This does not appear to in increase mortality or complications. - 19 mm SJM has low ΔP in small patients and is a durable long-term substitute. - Inverted AV can be used since the smallest MV is 23 mm. - MPS pts are at high risk during anaesthesia and induction. - Awake intubation is recommended with use of fibreoptic bronchoscope.
Tan (1992)	VI	34 F	18 mo history of palpitations and exertional dyspnoea	AS: ΔP 68 mmHg; MS: ΔP 12 mmHg; EF 63%	AV: 19 mm SJM; MV: 21 mm SJM (inverted AV); Root enlargement with pericardial patch	Transnasal intubation while upright with fibreoptic bronchoscope; AV cusps thick, mildly calcified, and fused; MV leaflets and chordae thickened.	Extubated 4 d PO; Emergency tracheostomy performed due to ARD; D/C 21 d PO; Required laryngotracheoplasty due to airway stenosis	
Tan (1992)	VI	21 F	Dyspnoea, orthopnoea, and reduced exercise tolerance	AV: 0.5 cm^2^, ΔP 40 mmHg; MV: ΔP 28 mmHg; Severe MR; EF 70%;	AV: 19 mm SJM; MV: 21 mm SJM (inverted AV); Root enlargement with a pericardial patch	Transoral ET intubation with fibreoptic bronchoscopy with mild sedation; AV cusps thick and commissures fused; MV thickened and stenotic; Chordae short and fused.	Extubated PO 2; Functioning prostheses, EF 60%; D/C 9 d PO	
Marek (2021) [99]	VII	32 M	Exertional dyspnoea and productive cough	AV degeneration and calcification; AS: 0.69 cm^2^; ΔP 58 mmHg; Moderate AR; Severe MV thickening and calcifications: 1 cm^2^; Moderate MR; LVH; chronic occlusion of RCA	ns; RCA bypass	Head reclining avoided during intubation; Uneventful surgery.	D/C 12 d PO; Normal echocardiogram at 6 mo F/U; No signs of HF at 12 mo	- Pt had relative lack of symptoms up to point of HF decompensation. - Restrictive pulmonary disease most likely caused by HF. - Cardiac causes of respiratory symptoms should be considered in MPS patients.

Abbreviations: ΔP = mean transvalvular pressure gradient; AR = aortic regurgitation; AS = aortic stenosis; AV = aortic valve; AVT = aortic valve thickening; CABG = coronary artery bypass graft; D/C = discharge; EF = ejection fraction; ERT = enzyme replacement therapy; ET = endotracheal; F/U = follow-up; HF: heart failure; IABP = intra-aortic balloon pump; LVH = left ventricular hypertrophy; MPS = mucopolysaccharidoses; MR = mitral regurgitation; MS = mitral stenosis; MV = mitral valve; MVT = mitral valve thickening; ns = none stated; NYHA = New York Heart Association functional classification; PAP = pulmonary artery pressure; PO = post-operative; RCA: right coronary artery; SJM = St Jude Medical mechanical prosthesis; TR = tricuspid regurgitation; TV = tricuspid valve.

**Table A3:** Description of case reports of mitral valve replacement in MPS patients.

Case	Type	Age/Sex	Presentation	Pre-op Findings	Device/Other Procedures	Surgical Notes	Outcome	Author Comments
Brazier (2015) [68]	I-HS	24 F	Paroxysmal dyspnoea, and stridor; On ERT	Severe MS: 0.9 cm^2^, ΔP 14.5 mmHg; 8 cm LA appendage aneurysm; PAP 50 mmHg	17 mm SJM (inverse AV); LA aneurysm resection	High MAP maintained due to concern for spinal ischaemia from previous occipitocervical fusion	Small paravalvular leak. Tracheostomy removed 15 d PO; D/C 37 d PO; NYHA Class I at 10mo F/U	- Small and rigid annulus. - Poor tissue quality. - Need for MDT preop planning for fibreoptic intubation and planned open tracheostomy. - Steroids used to prevent post-extubation laryngeal oedema.
Encarnacion (2017) [72]	I-HS	32 F	Reduced functional capacity; Previous Konno root enlargement; 21 mm SJM AVR; PO 42 d redo for suspected endocarditis; On ERT	MV tethered and thickened; MS: ΔP 12 mmHg; MR; EF 60%	25 mm SJM	Uneventful	PO 6 d echo: well-seated valve, ΔP 7.7 mmHg; AV well seated, ΔP 15 mmHg;	- Example of disease progression in spite of ERT. - Unknown if ERT can alter progression of valve disease. - VP shunt in situ: avoid entering pleural space to prevent infection. - Prudent to use mechanical valve to prevent future reintervention.
Manna (2021) [72]	I-HS	44 M	MV restenosis; Previous AVR, MV commissurotomy; On ERT	ns	ns; “Toilet of aortic prosthesis”; Removal of subvalvular fibrous tissue; AVN ablation pacing	ns	Normal life at 1 yr F/U.	- One of the longest living MPS I pts. - History suggests valve disease is stabilised or unresponsive to ERT. - MPS patients will likely need valve surgery because of longer lifespan since ERT.
Fischer (1999) [100]	I-S	35 M	Severe MS; SJM AVR 12 years previously.	MV and chordae thickening and calcification; MS: 1.2 cm^2^, ΔP 10 mmHg; Mild MR; Aortic prosthesis: ΔP 41 mmHg; EF 60%; PAP 55 mmHg	SJM	Extensive irregular thickening and calcification of MV and chordae.	Improved cardiopulmonary function at 6 mo F/U	- Small valve annulus - Difficulty in inserting adult prosthesis
Kitabayashi (2007) [65]	I-S	41 F	Exertional dyspnoea, NYHA III	Severely thickened and fused chordae, leaflets and papillary muscles; Severe MS: 0.90 cm^2^; Large LA: 49 mm; Mild AS and TR; EF 66%; PAP 52 mmHg	20 mm ATS	Difficult intubation with macroglossia and short neck; Difficulty identifying leaflet/annulus border; Reinforcement of suture line with equine pericardial patch between valve ring and LA wall; Annulus hard/not pliable.	ECMO/IABP due to severe diastolic LV dysfunction; Removed 3 d PO; Good valve function at 11 mo F/U.	- Small valve insertion due to small body size and annulus - Poor tissue quality and annulus flexibility. - Equine pericardial patch may be useful adjunct to prevent valve dehiscence and leakage. - IABP useful for low diastolic dysfunction. - MPS causes multivalvular disease; Other lesions need to be monitored.
Bhattacharya (2005) [75]	II	28M	Acute HF precipitated by new onset AF; Chronic MS	MV commissural fusion, thickened leaflets with subvalvular involvement; MV: 0.95 cm^2^	23 mm SJM	Thickened leaflets and chordae;	PO IABP and adrenaline; Elective tracheostomy; prolonged PO ventilation due to persistent bibasal atelectasis; D/C 18 d; Good LV function at 18 mo F/U	- Intubation difficult due to macroglossia and short tracheal length. - LMA used and ET tube passed through it. - Surgery complicated by poor tissue quality, small chambers and small mediastinum. - No clear demarcation of annulus and valve leaflets. - Tracheostomy due to risk of obstruction from macroglossia - Preoperative planning is important.
Lee (2013) [101]	II	25 M	Severe dyspnoea, NYHA IV	Thickened MV leaflets and subvalvular structures, commissural fusion; Severe MS: 0.6 cm^2^, ΔP 27 mmHg; PAP 63 mmHg	25 mm SJM	ns	Stable condition at 1 yr F/U; Started on ERT	- Difficult to differentiate rheumatic MS and MS secondary to MPS by echocardiography. - Diffuse and general thickening of MV and subvalvular structures, restrictive motion of leaflets may suggest MPS.
Ribeiro (2014) [102]	III-A	6 F	Anasarca and pneumonia	Severe MR; rupture of chordae; LV dilation	Biological prosthesis	ns	Mild AR and normalised LV function; Died at 13 from aspiration pneumonia	-
Marwick (1992) [103]	VI	25 F	Progressive exertional dyspnoea	MV rigidity, with commissural fusion; MS: 0.83 cm^2^, ΔP 18 mmHg	2 M Starr–Edwards 6120	MV: thickened, nodular, and calcified	Moderate AS at 3 yr F/U; Improved functioning	- Valve involvement similar to rheumatic fever with nodular thickening along free margin and shortening of chordae. - Cardiac involvement should be considered in progressive dyspnoea.
Bell (2018) [56]	VI	29 F	Symptomatic severe MV disease; On ERT	Severe MR; MV thickening, prolapsed leaflets; MV ΔP 10 mmHg; PAP 25 mmHg	21 mm Medtronic Standard pivot (inverted AV prosthesis)	ns	D/C 6 d PO; No obvious regurgitation at 10 mo F/U	- Mitral annular tissue is more friable. - Anchoring of prosthesis is more difficult. - Felt pledgets can be used to reinforce periprosthetic sutures. - Small annulus may require an inverted aortic prosthesis. - Consider extra-annular patches and mitral ring enlargement.

Abbreviations: ΔP = mean transvalvular pressure gradient; AR = aortic regurgitation; AS = aortic stenosis; AV = aortic valve; AVT = aortic valve thickening; D/C = discharge; EF = ejection fraction; ERT = enzyme replacement therapy; ET = endotracheal; F/U = follow-up; IABP = intra-aortic balloon pump; LA = left atrium; LMA = laryngeal mask airway; LV = left ventricle; MPS = mucopolysaccharidoses; MR = mitral regurgitation; MS = mitral stenosis; MV = mitral valve; MVT = mitral valve thickening; ns = none stated; NYHA = New York Heart Association functional classification; PAP = pulmonary artery pressure; PO = post-operative; SJM = St Jude Medical mechanical prosthesis; TR = tricuspid regurgitation.
